# Melatonin Enhances the Low-Calcium Stress Tolerance by Regulating Brassinosteroids and Auxin Signals in Wax Gourd

**DOI:** 10.3390/antiox13121580

**Published:** 2024-12-22

**Authors:** Jingjing Chang, Xuemei Zhu, Yixuan Lian, Jing Li, Xiao Chen, Zhao Song, Lei Chen, Dasen Xie, Baige Zhang

**Affiliations:** Key Laboratory for New Technology Research of Vegetable, Vegetable Research Institute, Guangdong Academy of Agricultural Science, Guangzhou 510640, China; changjing0710@nwsuaf.edu.cn (J.C.); 2063826277@stu.scau.edu.cn (X.Z.); 52308031059@fafu.edu.cn (Y.L.); lijing@gdaas.cn (J.L.); chenxiao@gdaas.cn (X.C.); songzhao@gdaas.cn (Z.S.); chenlei@gdaas.cn (L.C.); xiedasen@gdaas.cn (D.X.)

**Keywords:** low-Ca stress, melatonin, brassinosteroid, auxin, wax gourd

## Abstract

In plants, calcium (Ca) serves as an essential nutrient and signaling molecule. Melatonin is a biologically active and multi-functional hormone that plays an important role in improving nutrient use efficiency. However, its involvement in plant responses to Ca deficiency remains largely unexplored. This study aimed to assess the effects of melatonin on Ca absorption, the antioxidant system, and root morphology under low-Ca (LCa) stress conditions, as well as to identify key regulatory factors and signaling pathways involved in these processes using transcriptome analysis. Under LCa conditions, wax gourd seedling exhibited significant decreases in Ca accumulation, showed inhibition of root growth, and demonstrated the occurrence of oxidative damage. However, melatonin application significantly enhanced Ca content in wax gourd seedlings, and it enhanced the absorption of Ca^2+^ in roots by upregulating Ca^2+^ channels and transport genes, including *BhiCNGC17*, *BhiCNGC20*, *BhiECA1*, *BhiACA1*, and *BhiCAX1*. Furthermore, the application of exogenous melatonin mitigated the root growth inhibition and oxidative damage caused by LCa stress. This was evidenced by increases in the root branch numbers, root tips, root surface area, and root volume, as well as enhanced root vitality and antioxidant enzyme activities, as well as decreases in the reactive oxygen species content in melatonin treated plants. Transcriptome results revealed that melatonin mainly modulated the brassinosteroids (BRs) and auxin signaling pathway, which play essential roles in root differentiation, elongation, and stress adaptation. Specifically, melatonin increased the active BR levels by upregulating *BR6ox* (a BR biosynthesis gene) and downregulating *BAS1* (BR degradation genes), thereby affecting the BR signaling pathway. Additionally, melatonin reduced IAA levels but activated the auxin signaling pathway, indicating that melatonin could directly stimulate the auxin signaling pathway via an IAA-independent mechanism. This study provides new insights into the role of melatonin in nutrient stress adaptation, offering a promising and sustainable approach to improve nutrient use efficiency in wax gourd and other crops.

## 1. Introduction

Calcium (Ca) is a vital nutrient for plants and has dual roles as a structural component of cell wall and membranes and as a secondary messenger. As a structural component, it provides stability and integrity to the cell wall and cell membrane [1,2]. Thus, Ca deficiency in plants can lead to the degradation of cell walls and a decrease in cell membrane stability, disrupting the balance between reactive oxygen species (ROS) production and scavenging, ultimately causing oxidative damage [3]. It is well known that deficiency of Ca^2+^ often occurs in young developing tissues like leaves and fruits due to the limited remobilization of Ca^2+^ from old to young tissues through phloem [4]. The occurrence of Ca^2+^ deficiency disorders is a serious concern in horticulture crop production because it causes significant economic losses, such as blossom-end rot in tomato, bitter pit in apples, leaf tip burn in Chinese cabbage, cavity spot in carrots, and blackheart in wax gourd [5,6,7]. Ca uptake from the soil occurs through apoplast and plasma membrane cation channels of roots [8], meaning that the root is the first to perceive calcium deficiency stress. Meanwhile, Ca^2+^ is necessary to allow expansion during tip growth in root [9]. Research indicates that Ca deficiency impedes the elongation and growth of plant roots, inhibits lateral root development, accelerates root lignification, and reduces root vitality [10,11], which further hinders the plant’s absorption of Ca and other elements, thereby negatively affecting its growth, development, and yield.

The absorption of Ca by roots from the soil or solution mainly occurs through apoplastic and symplastic pathways [4,8]. The Ca^2+^ transported by the apoplastic pathway freely move following the water flow driven by transpiration until they encounter the Casparian strip in the endodermis, which blocks further apoplastic movement [8,12]. Thus, Ca^2+^ must enter the symplast to reach the vascular system before the root forms a Casparian strip. In symplastic pathway, Ca^2+^ crosses the plasma membrane of root cells via specific calcium transporters or channels, entering the cytoplasm [4,8]. This uptake pathway is mediated by several types of Ca^2+^ transporters and channels, such as cyclic nucleotide-gated channels (CNGCs), Ca^2+^-ATPases, and Ca^2+^/H^+^ antiporters, which maintain Ca^2+^ homeostasis and ensure efficient uptake and translocation within the plant [4,13]. However, most of these channels have been investigated in the context of their potential involvement in signaling pathways rather than their role in nutrition. Meanwhile, little research has been conducted to elucidate the mechanisms underlying plant root responses to Ca deficiency.

Melatonin (*N*-acetyl-5-methoxytryptamine), a conservative molecule, has been discovered in many species and organs that plays important regulatory roles in the growth, development, and stress response of plants [14,15,16]. Melatonin mainly improves plant growth and enhances stress tolerance by regulating photosynthesis, the source–sink relationship, redox regulation, and ionic homeostasis [14,15]. Research has shown that melatonin can effectively enhance plants’ absorption and utilization of mineral nutrients under stress, resulting in a 20% increase in nutrient use efficiency [17]. For example, exogenous melatonin treatment can significantly increase the contents of mineral elements such as potassium (K), phosphorus (P), sulfur (S), Ca, iron (Fe), and zinc (Zn) in corn [17]. Under low-K and salt stress conditions, melatonin promotes K accumulation in tomato roots to maintain the K^+^/Na^+^ ratio and alleviate stress damage [18,19]. Under nitrogen stress, exogenous melatonin regulates plant nitrogen metabolism by affecting nitrate reductase, glutamine synthetase, and glutamate synthase, alleviating growth inhibition [20,21]. The root system plays a pivotal role in nutrient acquisition by plants, while melatonin exhibits the ability to regulate root growth and development under nutritional stress [22]. Under cadmium stress, melatonin enhances the plant’s ability to absorb mineral elements by regulating the root architecture, thereby alleviating the toxic effects of cadmium on plants [23]. In *Arabidopsis*, melatonin promotes root growth to cope with nitrogen deficiency [17]. Although the crosstalk between the melatonin and Ca^2+^ signaling pathways has been reported in some abiotic stress responses and developmental processes in plant, there is currently a lack of direct evidence supporting the role of melatonin in regulating Ca nutrition.

Melatonin plays a complex regulatory role in various physiological processes in plants, and analyzing its signal transduction pathways and the function of key genes provides novel directions and insights for understanding its biological function in higher plants. Recently, with advances in omics sequencing and analytical techniques, research into the signal transduction mechanisms of melatonin has been gradually expanding. Among these, plant hormone signaling plays a crucial role in melatonin’s regulation of plant growth, development, and response to environmental stress, involving auxin (IAA), abscisic acid (ABA), jasmonic acid (JA), and gibberellins (GA) [24]. Melatonin interacts with plant hormones by promoting or inhibiting the expression of genes related to hormone synthesis or signaling. For example, melatonin promotes primary root growth through an auxin-dependent pathway, but it antagonizes cytokinin [25]. The germination of seeds is facilitated by melatonin through its regulation of the balance between ABA and GA, thereby antagonizing the inhibitory effects of ABA on seed germination [26]. Melatonin also regulates ethylene biosynthesis and the expression of various ripening factors (e.g., *ACO*, *ACS*, and *ERFs*), thereby extending fruit shelf life [27]. A lack of melatonin reduces the expression of the *DWARF4* gene, leading to a decrease in brassinosteroid (BR) content, which results in a semi-dwarf phenotype in rice [28,29]. Although the signal transduction mechanisms of melatonin in response to environmental stresses have been explored, the specific molecular mechanisms vary under different stress conditions. Currently, the molecular mechanisms underlying melatonin’s regulation of mineral nutrient stress signaling remain to be further studied.

Wax gourd (*Benincasa hispida* (Thunb.) Cogn) is a vegetable crop of significant importance in China, India, and other tropical countries in Asia; it is characterized by its long storage period, which plays a crucial role in ensuring the stable supply of vegetables throughout the year [30]. Ca is a crucial mineral element that affects the storage quality of fruits, and its deficiency can lead to the occurrence of blackheart disorder in wax gourd [7]. Previous research has mainly focused on improving Ca content by applying Ca fertilizers while neglecting the plant’s ability to absorb Ca, causing the wastage of Ca resources [25]. The present study was conducted to explore the role of melatonin in Ca absorption and root traits under low Ca conditions. By investigating melatonin’s influence on Ca uptake, the antioxidant defense system, and root morphological characteristics, this research aims to provide insights into how melatonin can alleviate the effects of Ca deficiency. Furthermore, the identification of key regulatory factors and signaling pathways through transcriptome analysis will deepen our understanding of the mechanisms by which melatonin mitigates nutrient stress. The results of this study will provide more effective and sustainable strategies for managing Ca nutrition in wax gourd and potentially in other crops.

## 2. Materials and Methods

### 2.1. Plant Materials and Growth Conditions

Wax gourd (*Benincasa hispida* (Thunb.) Cogn., cv. Tiezhu2) seeds were germinated on moist filter papers in an incubator at 30 °C after surface sterilization with 3% sodium hypochlorite (NaOCl) solution for 5 min and immersion in distilled water for 6 h. The germinated seeds were subsequently sown into pots filled with a mixture of peat/vermiculite (3/1, *v*/*v*). Seedlings were cultivated in a growth chamber with environmental conditions as follows: a photoperiod of 12 h, temperatures of 28/18 °C (day/night), humidity of 60–70%, and a photosynthetic photo flux density (PPFD) of 200 μmol m^−2^ s^−1^. The seedlings at first-true-leaf stage were transplanted into a container filled with 2 × Hoagland’s nutrition solution (Table 1). When the seedlings had two fully expanded true leaves, we selected them to use in experimental treatments.

### 2.2. Experimental Design

To investigate the potential involvement of melatonin in response to low-calcium stress (LCa), the plants were exposed to nutrition solutions with five different doses of Ca. Endogenous melatonin content and expression level of melatonin biosynthesis gene (*BhiCOMT1*) in roots were measured after one day of treatment. The treatments of five Ca doses were as follows: (1) Control, 4.0 mmol L^−1^ Ca; (2) 50% Ca, 2.0 mmol L^−1^ Ca; (3) 10% Ca, 0.4 mmol L^−1^ Ca; (4) 1% Ca, 0.04 mmol L^−1^ Ca; (5) 0% Ca, 0 mmol L^−1^ Ca. Calcium gradient nutrient solutions were prepared by replacing Ca(NO_3_)_2_ with equivalent amounts of NH_4_NO_3_. In the rest experiments of this study, the Ca concentration was 0.04 mmol L^−1^ (1%) in the LCa nutrient solution.

To examine the effect of rhizospheric application with melatonin on alleviating LCa stress, wax gourd roots were treated with different concentrations (0, 0.05, 0.15, 1.5, 15, and 30 μmol L^−1^) of melatonin by adding the required amount of melatonin stock solution into the LCa culture solution. Ten days after melatonin and LCa treatment, samples (roots and shoots) were taken to analyze dry matter content and calcium content. The 1.5 μmol L^−1^ amount of melatonin was used for the subsequent experiments.

For further analysis of the physiological mechanism and defense gene network of melatonin in response to LCa stress, wax gourd plants were divided into four experimental groups: control (CK, 4 mmol L^−1^ Ca), low-Ca treatment (LCa, 0.04 mmol L^−1^ Ca), and low-Ca + melatonin treatment (LCa+MT, 0.04 mmol L^−1^ Ca and 1.5 μmol L^−1^ melatonin). Root samples in plants were collected at 1 d to analyze genes transcript levels and high-throughput mRNA sequencing and at 10 d to measure Ca concentration and antioxidant system. The harvested samples were immediately frozen in liquid nitrogen and stored at −80 °C until use.

### 2.3. Melatonin Content Measurements

The extraction of melatonin was performed using acetone–methanol method [31], and its quantification was measured through enzyme-linked immunosorbent assay following the protocol described by Li et al., 2017 [32]. Briefly, 0.5 g root samples were extracted in 5 mL of extraction mixture (acetone: methanol: water, 89: 10: 1, *v*/*v*/*v*). The extract was centrifuged at 4 °C and 4500× *g* for 5 min. Then, 1 mL of 1% trichloric acid solution was added to the supernatant for protein precipitation. After centrifugation (10,000× *g*, 4 °C) for 15 min, the resulting supernatant was purified using a Sep-Pak C18 cartridge (Millipore, Milford, MA, USA) to isolate melatonin. Finally, an immunoassay kit (Shanghai Lanpai Biotechnology Co., Ltd., Shanghai, China) was employed following the manufacturer’s instructions to measure melatonin. Colorimetric recording was performed via Epoch Microplate Spectrophotometer (BioTek, South Burlington, VT, USA).

### 2.4. Analysis of Ca Content

Fresh wax gourd seedlings were sampled, washed three times with distilled water, and wiped using filter paper. Then, the root and shoot sections were separated, fixed at 105 °C for 30 min, and dried in a forced-circulation air oven at 65 °C until reaching a constant weight. The dry mass of roots and shoots was determined by weighing. The dried roots and shoots were ground into powder and then subjected to digestion with a mixture of perchloric acid–nitric acid (HClO_4_-HNO_3_) to determine Ca concentrations using an atomic absorption spectrophotometer (ZA3000, Hitachi, Tokyo, Japan). The Ca content was quantified by multiplying the dry mass with Ca concentration, resulting in a unit of mg DW.

### 2.5. Determination of Root Vitality

Root vitality was determined using the method of Clemensson-Lindell (1994) [33]. The fresh roots (0.3 g) were rins ed with distilled water and cut into small pieces (2 mm length). Then, they were incubated with 5 mL of 0.06 M Na_2_HPO_4_–KH_2_PO_4_ containing 0.6% (*w*/*v*) triphenyltetrazolium chloride (TTC) at 37 °C for 3 h in the dark. After incubation, the reaction was stopped by adding 1 M H_2_SO_4_. Then, the samples were washed twice with 5 mL distilled water and ground with 80% (*v*/*v*) acetone to extract triphenylformazan. The absorption at 520 nm was read to calculate the root vitality.

### 2.6. Determination of H_2_O_2_, O_2_^−^, and Malondialdehyde Content

H_2_O_2_ content in the root samples was measured as reported in Willekens et al., 1997 [34]. The production rate of superoxide anion (O_2_^−^·) was measured according to Elstner and Heupel (1976) [35]. Malondialdehyde (MDA) content was determined by 2-thiobarbituric acid (TBA) reaction reported by Hodges et al., 1999 [36]. 

### 2.7. Antioxidant Enzyme Extraction and Activity Assay

Wax gourd root materials (0.3 g) were homogenized on ice with 3 mL of 25 mM HEPES buffer (pH 7.8) containing 0.2 mM EDTA, 2 mM AsA, and 2% PVP. Subsequently, the homogenates were centrifuged at 4 °C for 20 min at 12,000× *g*. The resulting supernatants were collected for the determination of enzymatic activity using spectrophotometric methods. Superoxide dismutase (SOD) activity was assayed according to Stewart and Bewley (1980) method [37], which is based on the photochemical reduction of NBT. Peroxidase (POD) was assayed by measuring an increase in the absorbance of guaiacol oxidation at 470 nm following the procedure described by Cakmak and Marschner (1992) [38]. A decline at 240 nm was recorded to measure catalase (CAT) activity, which was measured using the method of Patra et al., 1978 [39]. 

### 2.8. Total RNA Isolation, Transcriptome Sequencing, and Real-Time Quantitative PCR Analysis

The total RNA was extracted from nine root samples using TRIzol reagent (Invitrogen, Carlsbad, CA, USA) according to the manufacturer’s protocol. RNA integrity was assessed using the Agilent Bioanalyzer 2100 system (Agilent Technologies, Santa Clara, CA, USA). Sequencing libraries were prepared using the NEBNext^®^UltraTM RNA Library Prep Kit for Illumina^®^ (NEB, Ipswich, MA, USA) in accordance with the manufacturer’s guidelines and were sequenced on the Illumina sequencing platform by BioMarker Biotechnology Co., Ltd. (Beijing, China). There were nine groups of raw readings, including root samples from the control group (CK1, CK2, CK3), low-Ca group (LCa1, LCa2, LCa3), and combination group of low Ca and melatonin (LCa+MT1, LCa+MT2, LCa+MT3). Clean reads were aligned to the reference genome of wax gourd (Wax gourd (B227) v1) using HISAT v2.1.0 software and utilized for subsequent analyses.

Differentially expressed genes (DEGs) were identified using DESeq2 v1.22.1/edgeR v3.24.3 [40]. The thresholds for significantly different expression were set at a false discovery rate (FDR) < 0.01 and |log_2_ foldchange| ≥ 1.5. The accuracy of transcriptome sequencing data was validated by performing Real-Time Quantitative PCR (RT-qPCR) analysis using Talent qPCR PreMix (TIANGEN, Beijing, China). PCR amplification was conducted utilizing a CFX96 Real-Time PCR Detection System (Bio-Rad, Hercules, CA, USA). The amplification program of PCR was set as follows: 95 °C for 3 min, followed by 40 cycles at 95 °C for 5 s and 60 °C for 15 s. Gene *BhiUBCP* (*Bhi07G001302*) was used as the internal reference. Gene-specific primers were designed using Primer Premier 6 (Supplementary Table S1).

### 2.9. Determination of Endogenous Brassinosteroids and Indole-3-Acetic Acid Contents

Fresh root samples were collected, immediately frozen in liquid nitrogen, ground into powder (30 Hz, 1 min), and stored at −80 °C for measuring brassinosteroids (BRs), indole-3-acetic acid (IAA), and methyl indole-3-acetate contents. For the extraction of BRs, a total of 50 mg fresh sample was weighed into a 2 mL plastic microtube and dissolved in 1 mL HPLC-grade acetonitrile (ACN). The extract was supplemented with 10 μL internal standard mixed solution (10 ng/mL) for the quantification. The mixture was vortexed for 10 min and then centrifuged for 10 min at 12,000× *g* and 4 °C. The supernatant was transferred to 2 mL amber glass vials, followed by adding 200 µL of 4-(N,N-dimethylamino) phenylboronic acid (4-DMAPBA). The reaction solution was vortexed, incubated at 75 °C for 1 h, and evaporated to dryness under nitrogen gas stream. It was then redissolved in 100 µL ACN and filtered through a 0.22 μm membrane filter [41]. The separation of BRs was achieved on a Waters ACQUITY UPLC HSS T3 C18 column (1.8 µm, 100 mm × 2.1 mm id).

The extractions of indole-3-acetic acid (IAA) and methyl indole-3-acetate were performed as described previously [42]. Briefly, 50 mg of fresh samples was weighed into a 2 mL microtube and dissolved in 1 mL methanol/water/formic acid (15:4:1, *v*/*v*/*v*), followed by overnight incubation. After adding 10 μL internal standard mixed solution (100 ng/mL), the mixture was vortexed for 10 min and centrifugated for 5 min (12,000× *g*, 4 °C). The supernatant was transferred to a clean plastic microtube, followed by evaporation to dryness and dissolution in 100 µL 80% methanol (*v*/*v*). The resulting solution was then filtered through a 0.22 μm membrane filter for further LC-MS/MS analysis.

The contents of BR, IAA, and methyl indole-3-acetate were detected by MetWare (http://www.metware.cn/, accessed on 3 January 2024) based on the AB Sciex QTRAP 6500 LC-MS/MS platform.

### 2.10. Statistical Analysis

The experimental design employed a completely randomized layout with three independent biological replicates. Each data value was expressed as the mean ± standard deviation (SD) from three replicates. The statistical analysis was conducted using one-way analysis of variance (ANOVA) followed by Turkey’s test. A value of *p* < 0.05 was considered to define significant differences between treatment means.

## 3. Results

### 3.1. Exogenous Melatonin Increases Ca Content in Wax Gourd Plants Under LCa Condition

**Figure 1:**
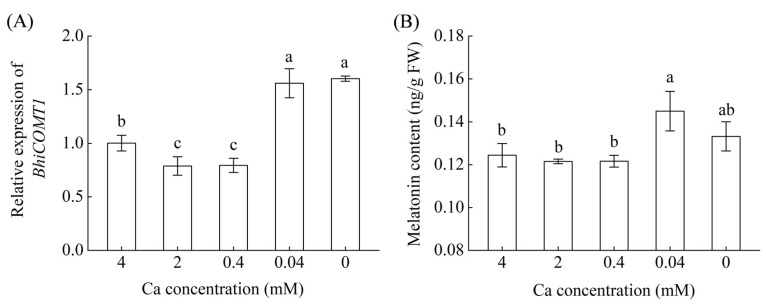
Effects of varied Ca concentrations on the relative expression of *BhiCOMT1* (**A**) and endogenous melatonin levels (**B**) in wax gourd roots. Wax gourd seedlings at the three-leaf stage were transferred to nutrient solutions containing varying Ca concentrations at 0, 0.04, 0.4, 2, and 4 mM for 1 d. Data in figures represent the means ± standard deviation (SD) of three replicates. Different letters represent significant differences at *p* < 0.05. *BhiCOMT1*: cafeic acid *O*-methyltransferase in wax gourd (*Benincasa hispida* (Thunb.) Cogn). FW: fresh weight.

**Figure 2:**
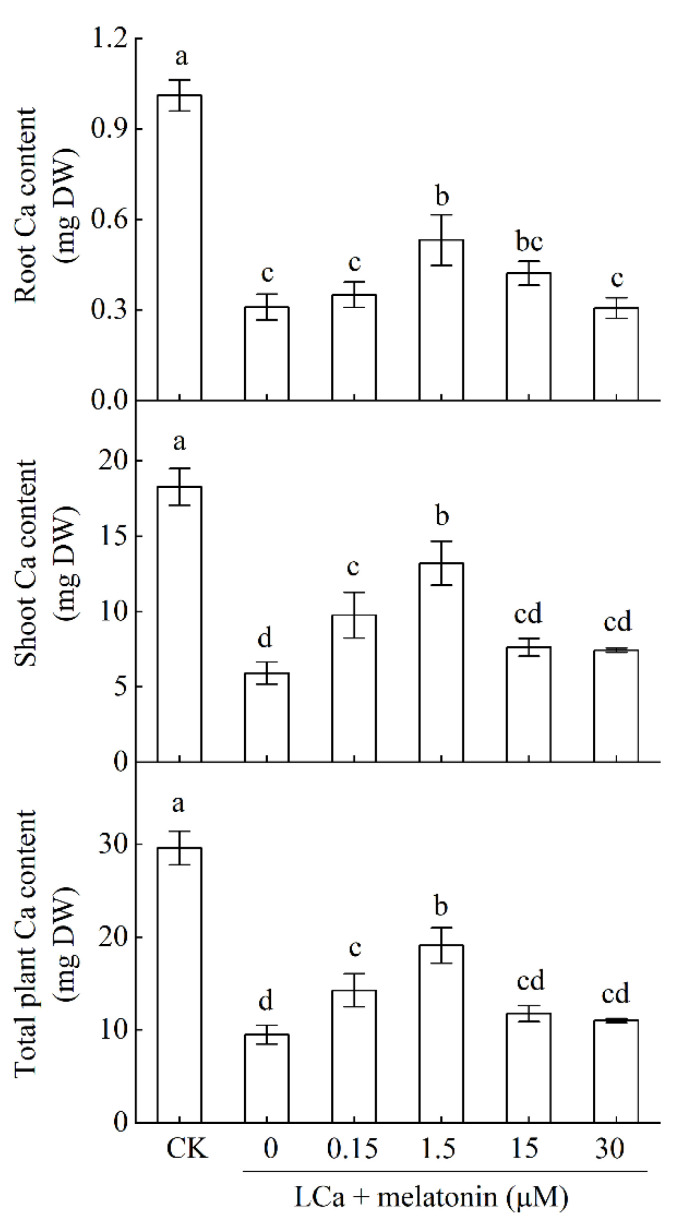
Selection of the optimal melatonin concentration to enhance Ca absorption under LCa stress. Wax gourd seedlings at three-leaf stage were subjected to a LCa nutrient solution at a concentration of 0.04 mM and treated with varying concentrations of melatonin (0, 0.15, 1.5, 15, and 30 μM) in the rhizosphere for 10 d. The root and shoot samples were collected for measuring Ca contents. Data in figures represent the means ± standard deviation (SD) of three replicates. Different letters represent significant differences at * p * < 0.05. DW: dry weight; CK: control.

The effects of varying Ca levels on melatonin biosynthesis in roots were first assessed to determine whether melatonin is involved in wax gourd’s response to Ca starvation. Cafeic acid *O*-methyltransferase (*BhiCOMT1*) is the key enzyme and rate-limiting enzyme for melatonin biosynthesis. As depicted in Figure 1, both the expression level of *BhiCOMT1* gene and melatonin content in roots were elevated at Ca levels lower than 1% in the nutrient solution, namely, 0.04 mM Ca and 0 mM Ca. The 0.04 mM Ca level subsequently was used as an LCa stress condition for further research. Under LCa conditions, the Ca contents of the roots and shoots decreased, while rhizospheric melatonin application at appropriate concentrations (0.15–30 μM) enhanced the Ca contents, with 1.5 μM proving most effective (Figure 2). Notably, wax gourd plants treated with 1.5 μM melatonin under LCa stress (LCa+MT) exhibited increases in their Ca content of 71.8%, 123.0%, and 101.2% in the roots, shoots, and entire plant, respectively, compared to LCa stress alone (LCa). These findings indicate that melatonin at an optimal concentration can promote Ca absorption in LCa conditions, whereas concentrations deviating from this optimum may diminish the promoting effect of melatonin on Ca uptake.

### 3.2. Effects of Rhizospheric Melatonin Application on Ca Absorption in Roots Under LCa Stress

**Figure 3:**
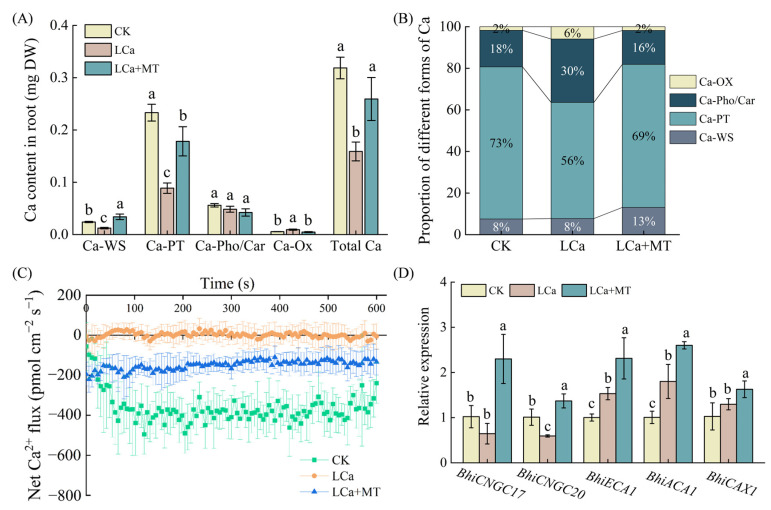
Effect of rhizospheric melatonin application on Ca dynamics in wax gourd roots under LCa stress. (**A**) Ca content of different forms in root; (**B**) proportion of different forms of Ca; (**C**) net Ca^2+^ flux; (**D**) relative expression of genes associated with Ca channels and transport. CK: control; LCa: low-Ca treatment; LCa+MT: low-Ca treatment + 1.5 μM melatonin treatment; DW: dry weight; Ca-WS: water-soluble Ca; Ca-PT: Ca-pectinate; Ca-Pho/Car: Ca phosphate/carbonate; Ca-Ox: Ca oxalate; *BhiCNGC17*: *cyclic nucleotide gated channel 17*; *BhiCNGC20*: *cyclic nucleotide gated channel 20*; *BhiECA1*: *calcium-transporting ATPase 1*; *BhiACA1*: *autoinhibited Ca^2+^-ATPase 1*; *BhiCAX1*: *vacuolar cation/proton exchanger 1*. Different letters indicate significant differences among treatments at *p* < 0.05.

Ca^2+^ is primarily absorbed in its cationic form by the root system from a soil or nutrient solution. To further evaluate the impact of melatonin on Ca absorption and utilization, we analyzed the contents and distributions of different Ca forms after LCa+MT and LCa treatments. As indicated in Figure 3, the roots exposed to LCa+MT treatment displayed significant changes in their Ca forms and proportions compared to the LCa treatment alone. The water-soluble Ca and Ca-pectinate levels in the LCa+MT-treated roots were 175.1% and 100.9% higher, respectively, than those in the LCa condition, while the Ca oxalate levels decreased by 49.7%. This shift resulted in an overall increase in the total Ca content under LCa+MT treatment (Figure 3A). Among all the Ca forms, Ca-pectinate was the predominant form with the highest proportion. The LCa treatment reduced the proportion of Ca-pectinate but increased the proportions of Ca phosphate/carbonate and Ca oxalate. Interestingly, the LCa+MT treatment restored the proportions of various Ca forms to the same level as the control (Figure 3B).

In this study, we detected the steady-state fluxes of Ca^2+^ in wax gourd roots to evaluate Ca absorption (Figure 3C). The Ca^2+^ in the control treatment showed an influx with an average flow rate of 379.4 pmol cm^−2^ s^−1^. After low-Ca treatment, the Ca^2+^ flux fluctuated around 0 pmol cm^−2^ s^−1^, indicating that the roots hardly absorbed Ca^2+^. Under low-Ca stress, after 24 h of treatment with 1.5 μM melatonin, the net Ca^2+^ influx fluxes were increased to 146.7 pmol cm^−2^ s^−1^. According to the data in Figure 3D, the LCa treatment downregulated the expression of the Ca channel genes *BhiCNGC17* and *BhiCNGC20* while upregulating the expression of *BhiECA1*, *BhiACA1*, and *BhiCAX1* compared to the control. Remarkably, the transcript levels of *BhiCNGC17*, *BhiCNGC20*, *BhiECA1*, *BhiACA1*, and *BhiCAX1* were upregulated by 3.6-, 2.3-, 1.4-, 1.5-, and 1.3-fold in LCa+MT-treated roots, respectively, compared to those in the LCa treatment alone. These results clearly indicate that melatonin promotes the absorption of Ca in roots under LCa conditions.

### 3.3. Effects of Rhizospheric Melatonin Application on Root Growth and Antioxidant Defense Under LCa Stress

**Figure 4:**
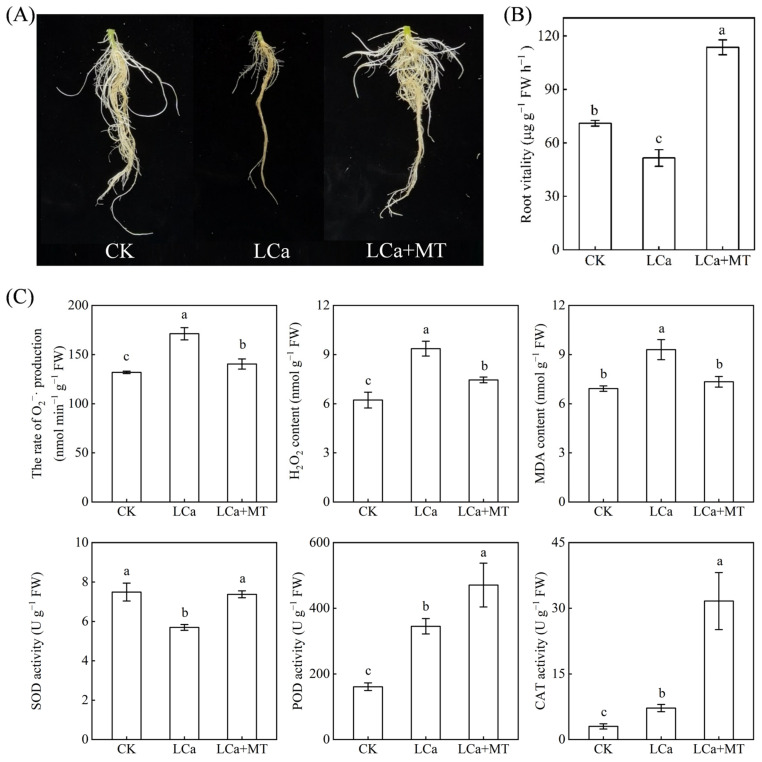
Effects of rhizospheric melatonin application on antioxidant system in root under LCa stress. Wax gourd seedlings at three-leaf stage were transferred to a low-Ca nutrient solution with concentrations of 0.04 mM and treated with 1.5 M melatonin in the rhizosphere for 10 d. (**A**) Root phenotype. (**B**) Root vitality. (**C**) Oxidative stress and antioxidant enzyme activities in roots. Data are means of three replicates (±standard deviation—SD). Different letters represent significant differences at *p* < 0.05. CK: control; LCa: low-Ca treatment; LCa+MT: low-Ca treatment + 1.5 μM melatonin treatment; FW: fresh weight.

As depicted in Figure 4A and Figure S1, LCa stress suppressed root growth and development, whereas the LCa+MT treatment alleviated the adverse effects of LCa on root growth. The data show that LCa treatment reduced the root branching numbers, root tip numbers, root surface area, and root volume, while these indicators in LCa+MT treatment increased by 58.7%, 76.7%, 27.4%, and 35.2%, respectively, compared to LCa, with no significant difference from the CK (Supplementary Figure S1). The root vitality directly affects its ability to absorb nutrients. Compared to the control (CK), the root vitality decreased by 37.8% under LCa treatment but increased by 60.0% under LCa+MT treatment (Figure 4B). The results from Figure 4C indicate that LCa led to oxidative stress in the roots of wax gourd, as evidenced by the accumulation of reactive oxygen species (ROS), including O_2_^−^·and H_2_O_2_, and an increase in the MDA content (Figure 4C). Antioxidant enzymes play pivotal roles in the defense against oxidative stress. The activities of peroxidase (POD) and catalase (CAT) were clearly enhanced under LCa stress, while the superoxide dismutase (SOD) activity was inhibited. Notably, the LCa+MT treatment boosted the activities of those antioxidant enzyme in the roots compared to the LCa-treated plants. For instance, the activities of SOD, POD, and CAT in the LCa+MT-treated roots increased by 29.5%, 36.4%, and 340.1%, respectively, compared to the LCa treatment.

### 3.4. Transcriptome Analysis of Root Response to Melatonin and LCa Stress

**Figure 5:**
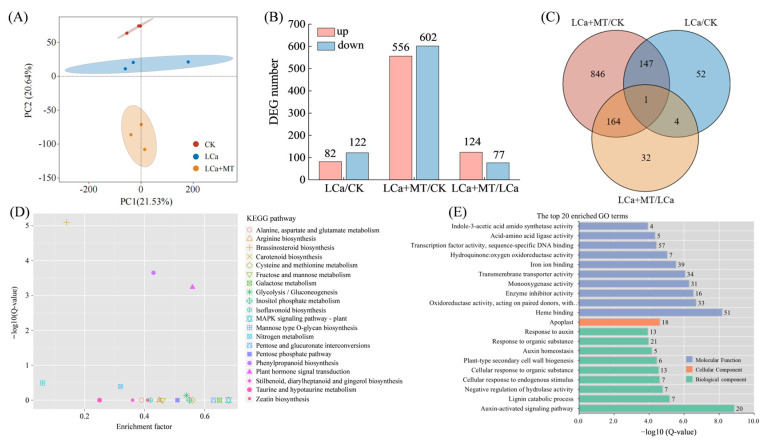
Analysis of differentially expressed genes induced by melatonin and/or LCa stress based on mRNA-seq. Wax gourd seedlings at three-leaf stage were transferred to a low-Ca nutrient solution with concentrations of 0.04 mM and the rhizosphere was treated with 1.5 M melatonin. Root samples were taken at 24 h for mRNA sequencing. (**A**) Principal component analysis (PCA); (**B**) the number of DEGs; (**C**) Venn diagram of DEGs; (**D**) KEGG pathway analysis of DEGs. The X and Y axis represent enrichment factor and −log_10_(Q-value), respectively. (**E**) GO terms of DEGs classified as biological, cellular, and molecular functions. The X and Y axis represent −log_10_(Q-value) and pathway names, respectively. The digits in the columns indicate the DEGs numbers for each pathway. CK: control; LCa: low-Ca treatment; LCa+MT: low-Ca treatment + 1.5 μM melatonin treatment.

Transcriptomic sequencing of roots was performed to study the effects of melatonin and low-Ca stress on the transcriptional profiles. Detailed statistics of the sequencing data and mapping results are provided in Supplementary Table S2 and S3, respectively. Principal component analysis (PCA) indicated distinct clustering of each sample group (Figure 5A). The criteria for differentially expressed genes (DEGs) were set as a fold change (FC) ≥ 1.5 and a false discovery rate (FDR) < 0.01. The analysis revealed 204 DEGs (82 up- and 122 downregulated) in the LCa/CK comparison, 1158 DEGs (556 up- and 602 downregulated) in the LCa+MT/CK comparison, and 201 DEGs (124 up- and 77 downregulated) in the LCa+MT/LCa comparison (Figure 5B). A Venn diagram revealed that only one DEG was common among all the comparisons, and 52 DEGs, 846 DEGs, and 32 DEGs were uniquely in the LCa/CK, LCa+MT/CK, and LCa+MT/LCa comparisons, respectively (Figure 5C).

Analyses of the Kyoto Encyclopedia of Genes and Genomes (KEGG) and the Gene Ontology (GO) were performed to elucidate the functions and biological processes commonly present in DEGs. Enrichment of the DEGs in the KEGG pathways and GO terms are shown in Figure 5D,E, displaying the top-20 enriched pathways and terms based on the smallest Q-values. The result in the KEGG pathway showed that the Q-values of the three pathways were less than 0.01, including brassinosteroid biosynthesis, phenylpropanoid biosynthesis, and plant hormone signal transduction, indicating significant enrichment of the DEGs in these three pathways. The GO analysis revealed that the top-20 GO terms were mainly enriched in the biological process and molecular function. Within the molecular function category, the majority of DEGs were associated with catalytic activities, transporter activities, and activities associated with bindings. In the biological process category, 36.5% of the DEGs were found to belong to auxin-related genes, including the auxin-activated signaling pathway, auxin homeostasis, and responsivity to auxin.

### 3.5. Transcriptome Profiles of Brassinosteroid Biosynthesis and Signaling-Related Genes

**Figure 6:**
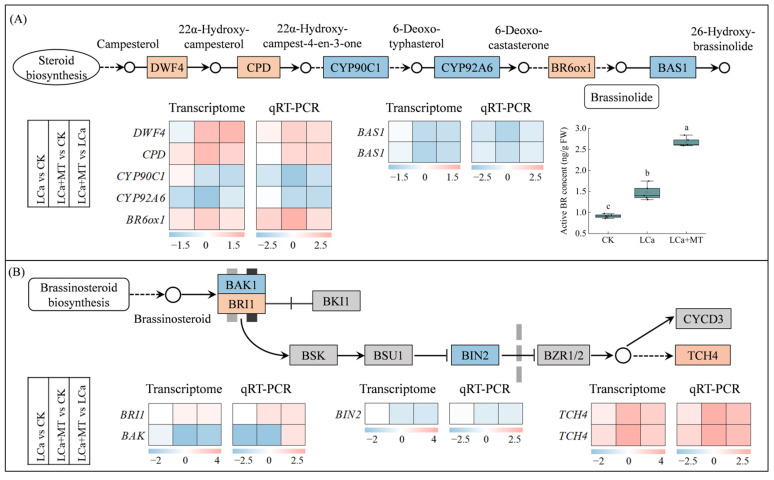
A heatmap of genes related to brassinosteroid biosynthesis (**A**) and signaling (**B**) pathway based on mRNA-Seq and RT-qPCR after rhizospheric melatonin application in LCa stress plants. The log2 fold-change values (Log_2_FC) for genes in LCa vs. CK, LCa+MT vs. CK, and LCa+MT vs. LCa comparisons were used to generate a heatmap. Detailed genetic information in the heatmap is present in Supplementary Table S1. Different letters represent significant differences at *p* < 0.05. CK: control; LCa: low-Ca treatment; LCa+MT: low-Ca treatment + 1.5 μM melatonin treatment; FW: fresh weight. “→” represents promotion; “
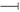
” represents inhibition; boxes represent genes; circles represent metabolites.

Crosstalk between the brassinosteroid (BR) and nutrient signals has been documented based on previous results [43]. In the current study, seven DEGs related to BR biosynthesis were identified, including *DWF4* (*Bhi06G000494*), *CPD* (*Bhi12G001778*), *CYP90C1* (*Bhi03G000681*), *CYP92A6* (*Bhi01G002193*), *BR6ox1* (*Bhi06G000253*), and two *BAS1* genes (*Bhi02G000875* and *Bhi02G000876*) (Figure 6A). Transcriptome data and RT-qPCR analysis revealed that only *BR6ox1* was upregulated under LCa stress alone, while the transcriptional abundances of the other genes showed no significant change. However, all these genes were regulated by melatonin under LCa stress (LCa+MT). For example, the expression levels of *DWF4*, *CPD*, and *BR6ox1* were upregulated, whereas *CYP90C1*, *CYP92A6*, and two *BAS1* genes were downregulated in the LCa+MT vs. CK and LCa+MT vs. LCa comparisons. Notably, *BR6ox1* was upregulated both in the LCa vs. CK and LCa+MT vs. CK comparisons, with increases of 2.6- and 5.5-fold, respectively, according to the results of RT-qPCR. The changes in transcription levels ultimately altered the levels of active BR in the LCa and LCa+MT treatments, with their contents exhibiting 1.2- and 3.1-fold increases, respectively, compared to the control.

Furthermore, five DEGs were identified in the BR signaling pathway (Figure 6B). Among them, two *TCH4* genes (*Bhi06G001170* and *Bhi06G001171*) were upregulated by LCa stress, with further enhancement by melatonin. Gene *BRI1* (*Bhi03G001100*), a positive regulatory factor of the BR signaling pathway, was upregulated by melatonin under LCa stress. Conversely, the negative regulators *BAK* (*Bhi09G001997*) and *BIN2* (*Bhi01G001488*) exhibited downregulation in the LCa+MT treatment, with their expression levels being 0.32- and 0.58-fold lower than those in the LCa treatment, respectively.

### 3.6. Effect of Melatonin on Auxin Signaling-Related Genes Expression Under LCa Stress

**Figure 7:**
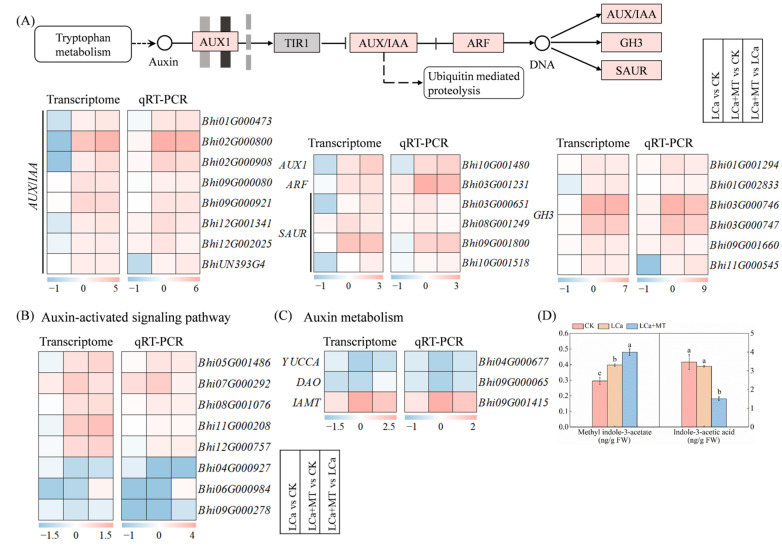
A heatmap of genes related to auxin signaling and metabolism pathway based on mRNA-Seq and RT-qPCR after rhizospheric melatonin application in LCa stress plants. (**A**) Auxin signaling pathway; (**B**) Auxin-actived signaling pathway; (**C**) Genes related to auxin metabolism; (**D**) Methyl indole-3-acetate and indole-3-acetic acid contents. The log2 fold-change values (Log_2_FC) for genes in LCa vs. CK, LCa+MT vs. CK, and LCa+MT vs. LCa comparisons were used to generate the heatmap. Specific genetic information is present in Supplementary Table S1. Different letters represent significant differences at *p* < 0.05. CK: control; LCa: low-Ca treatment; LCa+MT: low-Ca treatment + 1.5 μM melatonin treatment; FW: fresh weight. “→” represents promotion; “
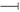
” represents inhibition; boxes represent genes; circles represent metabolites.

The interplay between melatonin and auxin is believed to govern plant root growth [24]. In the auxin signaling pathway, a total of 20 DEGs were identified, including one *auxin influx carrier 1* (*AUX1*), one *auxin response factor* (*ARF*), eight *auxin/indole-3-acetic acid* (*AUX/IAA*), six *indole-3-acetic acid-amido synthetase* (*GH3*), and four *small auxin-up RNA* (*SAUR*) (Figure 7A), which are key auxin response elements. The expression of these genes was generally downregulated under LCa stress, as revealed by transcriptome sequencing and RT-qPCR analysis. However, their expression levels exhibited a notable upregulation upon melatonin treatment under LCa stress (LCa+MT). For example, the expression levels of the *AUX1* and *ARF* genes in LCa+MT treatment were increased by 3.4- and 5.8-fold, respectively, compared to the LCa treatment, as determined by RT-qPCR. Meanwhile, eight DEGs were identified in the auxin-activated signaling pathway. Among them, the expression of four auxin efflux carrier components (*Bhi07G000292*, *Bhi08G001076*, *Bhi12G000757*, and *Bhi06G000984*) remained largely unaltered under LCa stress; however, they exhibited upregulation in response to the LCa+MT treatment with fold changes of 2.2, 2.3, 2.6, and 1.3 compared to the LCa treatment, respectively. The analysis of auxin metabolism revealed that melatonin treatment under LCa stress suppressed the expression of two crucial auxin biosynthesis genes (*YUCCA* and *DAO*) while enhancing the expression of the *indole-3-acetate O-methyltransferase* (*IAMT*) gene. Corresponding to these gene expression changes, melatonin treatment led to a reduction in the indole-3-acetic acid (IAA) level and an increase in the methyl indole-3-acetic acid content. These results indicate that exogenous melatonin may stimulate auxin signal transduction through an auxin-independent pathway under LCa stress in wax gourd roots.

## 4. Discussion

Recently, the involvement of melatonin in the nutritional stress response has received widespread attention. According to these studies, the exogenous application of melatonin exhibits potential for enhancing plant growth and improving plant tolerance towards nutrient stress [17]. Numerous studies have shown that Ca^2+^ signaling is involved in melatonin-regulated stress tolerance, growth, and development, including seed germination, leaf senescence, cold, and salt stress [23,26,44]; however, so far, little is known about the roles of melatonin involved in the LCa stress response.

### 4.1. Exogenous Melatonin at Appropriate Concentration Promotes Ca Accumulation in Wax Gourd Plants Under LCa Stress

In this study, the key gene *COMT1* involved in melatonin synthesis was upregulated, and the endogenous melatonin content was remarkably increased after LCa treatment in wax gourd (Figure 1). Previous studies have reported that endogenous melatonin biosynthesis is increased in common stress situations, such as cold, salt, or low potassium [18,23,32]. Meaningfully, the increase in endogenous melatonin content after LCa treatment suggests the possible involvement of melatonin in the response of wax gourd roots to LCa stress. Thus, we further focused on investigating the role of exogenous melatonin in wax gourd’s response to LCa stress. Our results indicated that exogenous melatonin application (1.5 μM) increased the accumulation of Ca in wax gourd plants (Figure 2), with its effect exhibiting dose dependence. These findings are in accord with those from previous investigations, indicating that appropriate concentrations of melatonin treatment can enhance stress tolerance, such as low temperature, salt, drought, and so on [18,32,45]. Under LCa stress, the Ca accumulations in the shoots and roots of plants treated with melatonin were higher than those of untreated plants (Figure 2), indicating that melatonin promotes absorption, transport, and accumulation of Ca, thereby influencing its distribution throughout the plant. Meanwhile, the measurement of steady-state Ca^2+^ fluxes and the expression analysis of Ca^2+^ channel and transport genes in root tip cells also revealed that under LCa stress, melatonin enhanced the absorption capacity of roots for Ca (Figure 3). For the next step, it is meaningful to investigate how melatonin regulates Ca absorption in the root system.

### 4.2. Exogenous Melatonin Enhances Ca Absorption Through Promoting Root Development and Mitigating Oxidative Stress Under LCa Stress

Plant root architecture plays a critical role in nutrient acquisition and is greatly influenced by soil nutrient availability. LCa stress can hinder the growth and development of roots, subsequently impacting the overall plant growth and yield [10,11]. In this study, LCa stress significantly reduced the root branching numbers, root tip numbers, surface area, volume, and root vitality (Figure 4 and Figure S1). These indices exhibited significant enhancement in plants that were treated with exogenous melatonin (Figure 4 and Figure S1). Numerous studies have already demonstrated the efficacy of exogenous melatonin in promoting root growth and elongation, enhancing the root absorption area and improving root vitality under abiotic stress conditions [46,47,48]. The results of this study further support the idea of a role played by melatonin in root morphology changes contributing to adaptation to stress conditions [49]. 

In plants, Ca exists in various forms, including water-soluble Ca, Ca-pectinate, Ca phosphate, and Ca carbonate, as well as Ca oxalate, whose contents and proportions will be changed by the supply levels of Ca [7,11]. Among these forms, Ca-pectinate plays a crucial role in maintaining cell wall integrity and facilitating cell-to-cell adhesion [50]. This study found that the main form of Ca in wax gourd roots is Ca-pectinate, which underwent the greatest decrease in content and proportion under LCa stress (Figure 3). Therefore, the decrease in Ca-pectinate content could cause instability of the cell wall structure, leading to the inhibition of root growth and damage caused by oxidative stress (Figure 4 and Figure S1) [7]. Exogenous melatonin treatment had the greatest effect on increasing the contents and proportions of Ca-pectinate, followed by water-soluble Ca. The action of melatonin can have dual effects: on the one hand, the higher Ca-pectinate content in the roots contributes to maintaining the structure and stability of cell walls, thereby reducing oxidative stress (Figure 3 and Figure 4); on the other hand, an increase in water-soluble Ca amount might facilitate intracellular movement of Ca^2+^, not only promoting Ca^2+^ signal transmission but also enhancing the transfer of free Ca^2+^ to the shoots (Figure 2). Furthermore, the robust antioxidant function of melatonin plays a crucial role in enhancing plant resistance to various stresses [22]. In this study, the addition of exogenous melatonin significantly decreased the MDA and ROS contents of roots and increased the SOD, POD, and CAT activities (Figure 4). These findings are consistent with previous research, indicating that exogenous melatonin mitigates membrane lipid peroxidation in plants and maintains membrane permeability under nutrient deficiency [47,48]. Collectively, it could be argued that the positive results were on account of the fact that melatonin enhanced the Ca absorption capacity of roots under LCa stress by regulating root development and bolstering antioxidant efficacy.

### 4.3. Melatonin Positively Regulates BR Synthesis and Signal Transduction Under LCa Stress

Numerous studies have established evidence that melatonin plays the regulatory roles in multiple physiological processes and stress tolerance by interacting with plant hormones or second messengers [14,24,51]. Therefore, there is a possibility of the existence of a signaling pathway responsible for the alterations in plant root system architecture and Ca uptake by melatonin. To study the signaling mechanism established by the regulatory role of melatonin in plants under LCa stress conditions, transcriptome sequencing was performed in LCa/CK, LCa+MT/CK, and LCa+MT/LCa environments (Figure 5). According to the KEGG pathway and GO analyses, the DEGs were mainly involved in the phytohormone biosynthesis and signaling pathways, especially in brassinosteroids and in auxin pathways (Figure 5).

BRs are steroid hormones that modulate cell elongation and division to regulate multiple aspects of plant growth and development. There is evidence indicating that BRs are essential for root differentiation and growth by establishing an increasing signaling gradient along the longitudinal axis of the root, which regulates cell division and elongation [52]. In the elongation zone of the root tip, Brassinazole-resistant 1 (BZR1), a BR signaling component, directly interacts with SHORT-ROOT (SHR) to repress the activity of downstream lignification-related genes, while BR signaling rapidly diminishes in the mature zone, inhibiting the interaction between BZR1 and SHR to initiate the lignification process [53]. This process is crucial for the selective uptake and transportation of water and nutrients by root tip tissue. Recent evidence has increasingly demonstrated that BRs are involved in the plant root’s response to nutrient stress. Zhang et al., 2021 [54] found that boron deficiency hinders BR accumulation by downregulating the transcription levels of *BR6ox1* and *BR6ox2*, thereby blocking the BR signaling pathway to inhibit root elongation in *Arabidopsis*. Phosphorus deficiency inhibits root growth through reducing BZR1 activity [55]. Here it was found that LCa stress also affected the BR biosynthesis and signaling pathway, diverging from other nutritional stresses in its ability to enhance the accumulation of active BR by 1.2-fold (Figure 6), which could be attributed to the specific role of Ca in cell wall stability and signaling. These results suggest that BR biosynthesis is activated as part of the plant’s response to Ca deficiency.

Melatonin regulates the semi-dwarf phenotype of rice by modulating the biosynthesis of BR [28,29]. In this study, melatonin increased the accumulation of active BR induced by LCa stress, which is attributed to the upregulation of key genes in the BR synthesis pathway by melatonin treatment, such as *DWF4*, *CPD*, and *BR6ox1*. BR6ox1 is a key enzyme in BR biosynthesis, and it is responsible for catalyzing critical steps in converting BR precursors into active forms [56]. The upregulation of *BR6ox1* under LCa stress (a 2.6-fold increase) was further enhanced by melatonin treatment (a 5.5-fold increase) (Figure 6A). This finding suggests that *BR6ox1* is particularly sensitive to LCa conditions and that melatonin amplifies this response, potentially to support root growth and maintain homeostasis under stress. In the BR signaling pathway, melatonin enhanced the expression of *BRI1*, a key receptor in BR signaling, further promoting the downstream effects of BRs. Simultaneously, melatonin downregulated the negative regulators *BAK* and *BIN2*, which usually suppress BR signaling by inhibiting the action of *BZR1*, which is the main BR-responsive transcription factor. This indicates that melatonin not only boosts BR biosynthesis but also amplifies BR signaling, allowing the plant to fully activate the BR-related responses necessary for adapting to LCa conditions.

### 4.4. Melatonin Positively Regulates Auxin Signal Transduction Through an Auxin-Independent Pathway Under LCa Stress

Auxin plays a pivotal role in the regulation of plant development, such as root morphology modulation, the inhibition of root elongation, and the promotion of lateral root formation [57]. Multiple signaling molecules crosstalk with auxin to regulate the development of plant roots under stress conditions, which could be an adaptive mechanism for plant survival [58,59]. Since melatonin and indole-3-acetic acid (IAA) are structurally related, and tryptophan is the common precursor, the interaction between them is often of interest to researchers. However, it largely remains controversial as to the relationship between melatonin and auxin. Some research suggested that melatonin increases the content of endogenous auxin by upregulating the expression of auxin synthesis genes, including YUCCA (YUC) flavin monooxygenase-like proteins (*YUC3*, *YUC4*, *YUC7*, and *YUC8*) [60]. The results of Liang et al., 2017 [61] indicated that melatonin shapes root architecture by directly or indirectly activating the auxin signaling pathway in rice. Another research investigation found that melatonin regulates root meristem by repressing auxin synthesis and polar auxin transport in *Arabidopsis* [62]. The current study showed that melatonin positively influenced the expression of several auxin signaling-related genes under LCa stress (Figure 7). This finding aligns with a previous study that demonstrated the regulatory role of melatonin in auxin signaling elements such as *Aux/IAA* (a key regulator of auxin gene expression), *ARF* (auxin response factor), and *SAUR* (small auxin upregulated RNA genes), which mediate the activation of growth processes [62]. Interestingly, while melatonin increased the expression of genes related to auxin signaling, it had a suppressive effect on the auxin biosynthesis genes *YUCCA* and *DAO*, leading to a reduction in the IAA levels (Figure 7). This result is consistent with previous research suggesting that melatonin can regulate root development processes through an auxin-independent signaling pathway in *Arabidopsis* [63]. These results indicated that under LCa stress, the role of melatonin may be independent of auxin signaling. Melatonin could enhance auxin signaling transduction without significantly changing the IAA level, which may suggest that melatonin can directly interact with key signaling factors such as Aux/IAA or ARF. This indicates that melatonin can directly enhance the auxin signaling response in plants under Ca deficiency, thereby facilitating adaptive growth strategies, such as enhanced root structure and functionality for improved nutrient absorption.

## 5. Conclusions

This study showed that LCa stress can result in an increase in the endogenous melatonin levels in wax gourd roots. Appropriate exogenous melatonin application not only facilitated the root absorption of Ca under LCa conditions but also conferred wax gourd root enhanced tolerance to LCa stress. These positive roles of melatonin can be attributed to optimized root structure and improved antioxidant defense system. Furthermore, transcriptomic analysis demonstrated that melatonin influenced both BR and auxin signaling, which are crucial for root growth and stress adaptation. Overall, the results indicate that treating with melatonin can be an effective strategy to enhance Ca uptake and mitigate the negative effects of Ca deficiency in wax gourd, providing a sustainable approach to improve nutrient use efficiency. These findings not only advance the understanding of melatonin’s role in nutrient stress regulation but also offer practical implications for improving crop resilience in nutrient-poor soils, contributing to sustainable agricultural practices.

## Figures and Tables

**Table 1:** Composition of nutrient solutions for wax gourd seedlings growth in a hydroponic system.

Composition	Normal Concentration	Low Ca Concentration
	mmol L^−1^	mmol L^−1^
N-NO_3_^−^	14	14
P-H_2_PO_4_^−^	1	1
K^+^	5	5
Ca^2+^	4	2, 0.4, 0.04, 0
Mg^2+^	2	2
S-SO_4_^2−^	2	2
	μmol L^−1^	μmol L^−1^
Fe-EDTA	15	15
Mn	10	10
B	25	25
Zn	5	5
Cu	1	1
Mo	0.5	0.5

## Data Availability

The data are contained within the article or Supplementary Materials.

## Supplementary Materials

The following supporting information can be downloaded at https://www.mdpi.com/article/10.3390/antiox13121580/s1: Table S1. Gene-specific primers used for RT-qPCR in this study. Table S2. Sequencing data Statistics. Table S3. Statistics on data mapping. Figure S1. Effects of rhizospheric melatonin application on root morphology under LCa stress.
