# Supplementary material for: Melatonin Enhances the Low-Calcium Stress Tolerance by Regulating Brassinosteroids and Auxin Signals in Wax Gourd
Source: Antioxidants (Basel). 2024 Dec 22;13(12):1580. doi: 10.3390/antiox13121580 (PMC11673479; doi:10.3390/antiox13121580)
Supplement: Supplementary file 1 [file antioxidants-13-01580-s001.zip › antioxidants-3331744-supplementary.pdf]

Supplementary Information

Supplementary Table S1 Gene-specific primers used for RT-qPCR in this study.

Supplementary Table S2 Sequencing data Statistics.

Supplementary Table S3 Statistics on data mapping.

Supplementary Figure S1 Effects of rhizospheric melatonin application on root morphology under LCa stress.

Supplementary Table S1 Gene-specific primers used for RT-qPCR in this study

Gene ID	Gene name	Forward sequence (5'-3')	Reverse sequence (5'-3')	Description
Bhi07G001302	*BhiUBCP*	ACCGACAGTTCGCTTTGTGTCT	GGGTCACACAGCAAGGACTGAA	Internal reference
Bhi01G002554	*BhiCOMT1*	ATGGTGCTGAAGACGGCGATTG	AGGATGTTGTAGCTGGCGAGCA	Caffeic acid *O*-methyltransferase 1
Bhi08g001608	*BhiCNGC20*	GCCAGTCTCCAAGTGGCTTTGT	AGAGCCAACGATATGACCAGCC	Cyclic nucleotide gated channel 20
Bhi02G000626	*BhiCNGC17*	TGGGAGAGGTGAGCTTGTGATG	CGGCTTCTTGTTGCGGGTATGA	Cyclic nucleotide gated channel 17
Bhi08G000566	*BhiECA1*	TGCCTGCTGACATGCGGGTATT	TCACTGCTTCACTCTCGCCTGT	Calcium-transporting ATPase
Bhi11G000346	*BhiCAX1*	TAATGGCTGTCATGGGCCTGCT	TCGAGAGTGCCAATTCCGACTT	Vacuolar cation/proton exchanger
Bhi09G002663	*BhiACA1*	GAGAATTTCGGCGACGTGAAGC	CCGATTCTTGACCAGCCAGCAA	Autoinhibited Ca^{2+}-ATPase 1
Bhi06G000494	*DWF4*	ACGTCGTCGTTAGCTTCACCAG	CGACTCCTTTGCTTCGATGCCA	Cytochrome P450
Bhi12G001778	*CPD*	ACACCGCAATGAAGGCAAGGAA	TGCTTTAGGCGAGTGGGTTAGG	3β,22alpha-dihydroxysteroid 3-dehydrogenase
Bhi03G000681	*CYP90C1*	AGGATGGTGCGTCTTGGCATCT	AGATTTCGAGCCTGGCGAGTTC	3-epi-6-deoxocathasterone 23-monooxygenase
Bhi01G002193	*CYP92A6*	CCTCTGTCGCCGCAAACTCAAT	TCCGACCACAACAGGGAATGAC	typhasterol/6-deoxotyphasterol 2alpha-hydroxylase
Bhi06G000253	*BR6ox1*	CGGCGCTCACAAGCTTATGAGA	TCGATTCCGGCAATCTGCTTCA	brassinosteroid 6-oxygenase
Bhi11G002164	*BAS1*	TTGGACCAATTGTCCGAGTCGC	GCCATCGCCTTCGAGCTGTTTA	PHYB activation tagged suppressor 1
Bhi11G002165	*BAS1*	GAAACCACTTGCTCGGCATTGC	AGCCTCTTCCGCCATCTCAACA	PHYB activation tagged suppressor 1
Bhi03G001100	*BRI1*	GCAGCACTTGGCATTGAAGGGA	CCAACGTCGCCGGTGAACTTAT	protein brassinosteroid insensitive 1
Bhi09G001997	*BAK*	TAGCCATTGGGACCGCTAAAGC	TGCCTCGATAGCCAGTGAGAGA	brassinosteroid insensitive 1-associated receptor kinase 1
Bhi01G001488	*BIN2*	GGGCTTGGGAATTTGACGCAGT	CGCCATGTTCAACCGAACCAGA	protein brassinosteroid insensitive 2
Bhi06G001170	*TCH4*	GAGATGGGCGAGGTCAGATGTT	TGACAGTGCCAGCAGAGTTTCC	xyloglucan:xyloglucosyl transferase TCH4

Bhi06G001171	*TCH4*	CAAAGGAGTGCCCTTCCCACAA	GGGCTTGGCTCCAATCTGTCTT	xyloglucan:xyloglucosyl transferase TCH4
Bhi01G000473	*AUX/IAA*	AGGGTTAGGGCTCAGTCTTGGA	ACAGCAACAGGTCTGCCAGAGA	
Bhi02G000800	*AUX/IAA*	TCAACCACTCTGCGGCATTTCG	ACAAGCCCATTCTCCCGTTCCT	
Bhi02G000908	*AUX/IAA*	GTCACGACCAGCCTCAACTCAT	CCCATCAGCCGCAACTTTAACG	
Bhi09G000080	*AUX/IAA*	CGGATCTTGCCCTTGCTCTTCA	ACGTCTCCAACAAGCATCCAGT	
Bhi09G000922	*AUX/IAA*	TGGCGTTCGTGAAAGTGAGCAT	ACATGGCAGCAAGAGCATCAGA	auxin/indole-3-acetic acid
Bhi12G001341	*AUX/IAA*	GGCGGCAAGGTTAATGGTGGTA	GTCAGCAGCTCGTTTAGTCCCA	
Bhi12G002025	*AUX/IAA*	CACCGAGCTTCGTCTTGGACTA	CCACCCAACAACCTGAGCCTTT	
BhiUN393G4	*AUX/IAA*	GGTTAAACCTCGATGCGACGGA	GGCCATCCCACAATTTGAGCCT	
Bhi10G001480	*AUX1*	CAGCAGAGACGACGACAACGAT	GCTACCTGGTTTGAAGCACAGC	auxin influx carrier
Bhi03G001231	*ARF*	CGGAGTCGTCATGTGGATGGAA	AGCAAGTGCGGCAGCTTCAAT	auxin response factor
Bhi03G000651	*SAUR*	TGGCTGCAATGAAGACGGTCTC	CAGGCTTTGGAACTGCGGATGA	
Bhi08G001249	*SAUR*	ACGGCAACGGTTTGTTGTGAGA	GTCCATCTCCGCCAACACATGA	small auxin-up RNA
Bhi09G001800	*SAUR*	CGGTGCTCAAGCAGCTACTCAA	TCGATCCTGACCCACGTAGACA	
Bhi10G001518	*SAUR*	TGCCTTCGCTACTCCTCAATGC	TCAGCCCTCTTAAGCAAAGCCA	
Bhi01G001294	*GH3*	CCGCAATTCCACTTCGTGAGGA	AACAACTCCGACGCCTTCTCCA	
Bhi01G002833	*GH3*	GCTCTGGAACTTCTGGTGGACA	AACTGGCCTTGCCATTAACCCA	
Bhi03G000746	*GH3*	ACTCGGTTCAGCAGAGGGTGTT	TCGCCATTGGCAATACGTTGGA	auxin responsive GH3 gene family
Bhi03G000747	*GH3*	ATCCGCTTCCTCCAGCTCAACT	AATTCCGCCAGCTCTGGATTCG	
Bhi09G001660	*GH3*	AGCATGTACGCTCAGCTCCTCT	AGAACCGGATGGCTCGGATGAA	

Bhi11G000545	*GH3*	CGCTGTGTTTGCCTCTGGTTTC	TCACTGCGTCACGAACTTCCTG	
Bhi05G001486	*ATLP*	TTGCTGTGCCACCACTGTCTTC	AGGATGGAGGCAATGGTGAGGT	Auxin transporter-like protein
Bhi07G000292	*AEX4*	TCTTGACGGCGGTGATTCCTCT	ATGGCGACAAAGCGGTTGATCC	Auxin efflux carrier component 4
Bhi08G001076	*AEX1*	CCACGGCCTTCAAACTTGACCA	TTCCACCGGCAGCCATCATAGA	Auxin efflux carrier component 1
Bhi11G000208	*PBR*	TCCTCCTGTTAATCGGCACCCA	ATGTGAACACCAGGCTCCACCT	Protein BREVIS RADIX
Bhi12G000757	*AEX3*	TGCTGAGATTGGAGACGACGGA	AGATTCGAAGGCCGAGGCGTTA	Auxin efflux carrier component 3
Bhi04G000927	*PBGE*	ACAAGCAACCAAGCTCTCCTGG	CGACTTTCGTCCTCGTCCATGT	Protein BIG GRAIN 1-like E
Bhi06G000984	*AEX5*	AGCTTCTCGCTCTCCACTCTCA	GTCCGTCGAAGTTCCAGCACAA	Auxin efflux carrier component 5
Bhi09G000278	*PBGB*	GTCCAACGCGAAGAACAGGTGA	GCCATAGAGGTGCGACGAACAA	Protein BIG GRAIN 1-like B
Bhi04G000677	*YUCCA*	GTATGATCGCACGCTCGGGTTT	ACATCCATTCCGTCAAGCTCCG	Indole-3-pyruvate monooxygenase YUCCA6
Bhi09G000065	*DAO*	AGGTCTTGTAGGCACCGAGTGA	GCTCTTCAGGCACAGCAATCGT	2-oxoglutarate-dependent dioxygenase
Bhi09G001415	*IAMT*	GCTCAGGCTCAACATGCGAGAT	AGCCGAGATCCACAACCACGAA	Indole-3-acetate O-methyltransferase

Supplementary Table S2 Sequencing data Statistics

Samples	Clean Reads	Clean Base	Q20 (%)	Q30 (%)	GC Content (%)
CK-1	19,416,067	5,806,697,570	97.8	93.69%	44.46%
CK-2	19,284,148	5,766,804,852	98.07	94.34%	44.46%
CK-3	22,118,273	6,613,848,272	98.26	94.81%	44.30%
LCa-1	22,044,526	6,588,619,310	98.09	94.39%	44.45%
LCa-2	20,473,393	6,120,000,156	98.2	94.69%	44.23%
LCa-3	21,034,993	6,291,934,400	98.19	94.68%	44.18%
LCa+MT-1	19,421,667	5,807,057,230	98.26	94.87%	44.50%
LCa+MT-2	20,709,726	6,193,480,302	98.32	95.02%	44.69%
LCa+MT-3	20,931,614	6,261,062,652	98.17	94.62%	44.35%

Supplementary Table S3 Statistics on data mapping

Samples	Total Reads	Reads mapped	Unique mapped	Multi mapped
CK-1	38,832,134	34,494,145 (88.83%)	33,752,962 (86.92%)	741,183 (1.91%)
CK-2	38,568,296	34,616,061 (89.75%)	33,893,939 (87.88%)	722,122 (1.87%)
CK-3	44,236,546	39,506,339 (89.31%)	38,674,693 (87.43%)	831,646 (1.88%)
LCa-1	44,089,052	39,707,847 (90.06%)	38,886,395 (88.20%)	821,452 (1.86%)
LCa-2	40,946,786	37,306,688 (91.11%)	36,531,045 (89.22%)	775,643 (1.89%)
LCa-3	42,069,986	38,650,603 (91.87%)	37,860,299 (89.99%)	790,304 (1.88%)
LCa+MT-1	38,843,334	34,508,995 (88.84%)	33,803,623 (87.03%)	705,372 (1.82%)
LCa+MT-2	41,419,452	34,421,574 (83.10%)	33,739,659 (81.46%)	681,915 (1.65%)
LCa+MT-3	41,863,228	37,676,765 (90.00%)	36,859,730 (88.05%)	817,035 (1.95%)

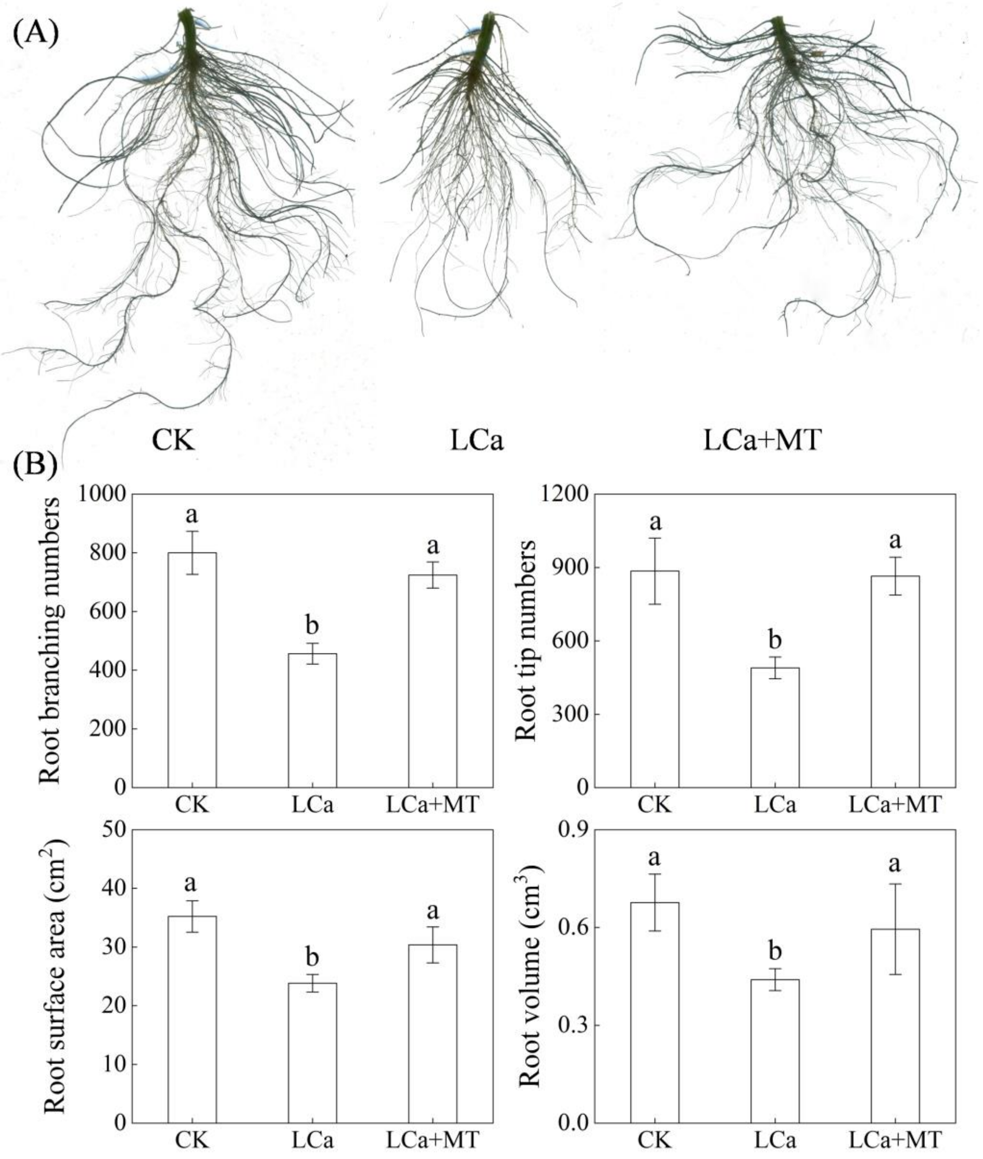

Supplementary Figure S1 Effects of rhizospheric melatonin application on root morphology under LCa stress. (A) Root morphology images obtained using the MICROTEK ScanMaker i800plus root scanner. (B) Root morphological indexes. Data in (B) represent the means ± standard deviation (SD) of three replicates. Different letters represent significant differences at $p<0.05$ according to Turkey's test.
